# Antibiofilm Activity of the Brown Alga *Halidrys siliquosa* against Clinically Relevant Human Pathogens

**DOI:** 10.3390/md13063581

**Published:** 2015-06-05

**Authors:** Alessandro Busetti, Thomas P. Thompson, Diana Tegazzini, Julianne Megaw, Christine A. Maggs, Brendan F. Gilmore

**Affiliations:** 1 School of Pharmacy, Queen’s University Belfast, 97 Lisburn Road, BT9 7BL Belfast, UK; E-Mails: a.busetti@qub.ac.uk (A.B.); tthompson08@qub.ac.uk (T.T.); dtegazzini01@qub.ac.uk (D.T.); j.megaw@qub.ac.uk (J.M.); 2 School of Biological Sciences, Medical Biology Center, 97 Lisburn Road, BT9 7BL Belfast, UK; E-Mail: c.maggs@qub.ac.uk

**Keywords:** *Halidrys siliquosa*, antimicrobial, antibiofilm, clinical strains, biodiscovery, extraction

## Abstract

The marine brown alga *Halidrys siliquosa* is known to produce compounds with antifouling activity against several marine bacteria. The aim of this study was to evaluate the antimicrobial and antibiofilm activity of organic extracts obtained from the marine brown alga *H. siliquosa* against a focused panel of clinically relevant human pathogens commonly associated with biofilm-related infections. The partially fractionated methanolic extract obtained from *H. siliquosa* collected along the shores of Co. Donegal; Ireland; displayed antimicrobial activity against bacteria of the genus *Staphylococcus*; *Streptococcus*; *Enterococcus*; *Pseudomonas*; *Stenotrophomonas*; and *Chromobacterium* with MIC and MBC values ranging from 0.0391 to 5 mg/mL. Biofilms of *S. aureus* MRSA were found to be susceptible to the algal methanolic extract with MBEC values ranging from 1.25 mg/mL to 5 mg/mL respectively. Confocal laser scanning microscopy using LIVE/DEAD staining confirmed the antimicrobial nature of the antibiofilm activity observed using the MBEC assay. A bioassay-guided fractionation method was developed yielding 10 active fractions from which to perform purification and structural elucidation of clinically-relevant antibiofilm compounds.

## 1. Introduction

The marine environment favors the formation of microbial biofilms on virtually all inanimate submerged surfaces [1]. In contrast, the majority of marine eukaryotic organisms, especially benthic, slow-moving or photosynthetic ones require their exposed biotic surfaces to remain relatively free from fouling [2,3]. Thus, they have evolved a plethora of antifouling strategies aimed at preventing the settlement and colonization of unwanted microbial pathogens and microfoulers [4] responsible for conditioning surfaces and providing cues for the settlement of macrofouling species such as barnacles [5]. The antifouling strategies adopted by marine organisms range from the purely mechanical, such as the production of mucus by fish [6], the nanopatterning of shark skin [7], or the periodical shedding (ecdysis) and replacement of the rigid exoskeleton by Crustaceans [8] to the biosynthesis of specific antifouling bioactives including a multitude of antimicrobials and quorum sensing inhibitors (QSIs) such as the renown halogenated furanones [9,10,11].

As all benthic marine photosynthetic organisms, brown algae are restricted to the euphotic zone where the fouling pressure is typically highest. As a result, the capacity to synthesize effective antifouling bioactives appears to have evolved as a principal antifouling strategy within this phylum [12,13,14,15]. Compounds produced by brown algae include major metabolites derived from isoprene (complex diterpenoids) [16], volatile compounds (cyclic or acyclic short-chain hydrocarbons (C8 or C11) arising from enzymatic conversion of long chain fatty acids), fucoidans, phlorotannins and fucoxanthins exhibiting antioxidant, antibiotic, antifungal, antiviral and anti-cancer activities [17]. *Halidrys siliquosa* is a brown alga found in rock pools and in the shallow subtidal waters of the Atlantic coasts of Europe, of the Baltic Sea, of Ireland and the British Isles [18]. Previous studies suggest this species relies heavily on an arsenal of chemicals to protect itself from grazing, fouling, pathogens and parasites. In fact the production of bioactives with anti-trypanosomal and anti-leishmanial activity [19], with antifouling activity against several marine bacteria [20] and displaying antimicrobial activity against some human pathogens [19,21,22] has been reported making this organism an ideal candidate for the isolation and characterization of bioactive compounds displaying antimicrobial or antibiofilm activity against clinically relevant human pathogens commonly associated with biofilm-related infections, especially ones displaying resistance to current antibiotics.

The aetiology of a significant number of acute and chronic human infections has been associated with the biofilm mode of growth of pathogenic bacteria [23]. In fact, current estimates suggest that the majority of human infections involve biofilms [24]. Within a biofilm, bacteria are provided with a greater degree of protection against challenging environmental conditions, natural and synthetic antimicrobials, chemical insults, mechanical removal, bacteriophages, external predation and elements of the body’s immune system such as leukocytes [25,26,27,28,29,30,31,32,33,34,35]. The successful formation of a biofilm within a human host often results in the development of a chronic, untreatable infection characterized by an elevated tolerance to conventional antibiotic treatment [36] and with an established capacity for evading host immune detection and response [37]. In fact, biofilm associated infections often fail to respond to standard antimicrobial therapy based on classical susceptibility studies using planktonic cultures (such as the minimal inhibitory concentration (MIC) and minimal bactericidal concentration (MBC)) and concentrations of antibiotics up to 100–1000 fold higher than those necessary to treat planktonically growing bacterial cultures are often required to completely eradicate the same bacteria growing in biofilms [38,39].

Microbial biofilms provide a favourable environment for the intra- and inter-specific horizontal transmission of genetic elements with the consequent dissemination of antimicrobial resistance (AMR) genes [40,41]. In the clinical environment a clear relationship between antimicrobial use and the emergence of multiresistant strains has been observed [42,43], severely undermining the efficacy of previously successful courses of treatment for both acute and chronic infections. For example, *Staphylococcus aureus* has gradually re-emerged as a clinically relevant pathogen due to its resistance to antibiotics and the increased availability and use of indwelling medical devices [43,44,45]. Multi-resistant *S. aureus* (MRSA) infections in the US have a crude mortality rate of 25% along with long hospitalizations periods [46,47]. *S. aureus* biofilm-related infections are currently involved in the majority of cases of Osteomyelitis, are often associated to chronic wound infections (such as diabetic foot ulcers, venous stasis ulcers and pressure sores) and represent the major cause of infection and failure of indwelling medical devices [45]. In the nosocomial environment, *S. aureus* biofilm infections are also commonly associated to the use of stents, ventilators, urinary and intravenous catheters, infusion pumps, mechanical heart valves, aspirators, pacemakers, stitch materials, ear and central nervous system shunts and cosmetic surgical implants [48] and can generally occur anywhere the skin barrier is compromised and bacteria can be introduced through a hematogenous route or through direct exposure during surgery [49].

Within the past two decades, the growing costs and efforts required to develop and market novel antibiotics has caused many major pharmaceutical companies to completely exit this field and focus their research efforts on products unlikely to lose their effectiveness over a short period of time such as antidepressants, statins, and anti-inflammatory medications. As a consequence there has been a continuous decrease in the number of new antibacterial drugs approved for marketing globally with an 88% drop in the approval of novel systemic antibiotics since the mid-1980s [50]. This scenario points to the likelihood of a substantial increase in morbidity and mortality worldwide, justifying and necessitating renewed interest in research aimed at the discovery of novel antibiofilm compounds and strategies focused on countering the emergence of antimicrobial resistance. An example of one such promising strategy is the inhibition of QS (QSI), the cell-to-cell signaling system responsible for regulating the expression of genes necessary for virulence factor production, for the production of products required for bacteria-host interactions and for the regulation of biofilm development [51,52,53,54,55,56,57,58,59,60]. The QSI approach aims at disarming rather than killing pathogens whilst rendering them more susceptible to conventional antimicrobial treatments [61] and to the host immune responses [62]. Moreover, as QS is not involved in mechanisms essential for the survival of bacteria, its inhibition is unlikely to produce a harsh selective pressure apt to cause the emergence of resistance [63].

The immense chemical diversity of marine algae provides a rich potential for the necessary, upcoming concerted global effort required for the discovery of novel antimicrobials and strategies apt to tackle bacterial infection and the emergence and diffusion of AMR in the 21st century.

## 2. Results

**Figure 1:**
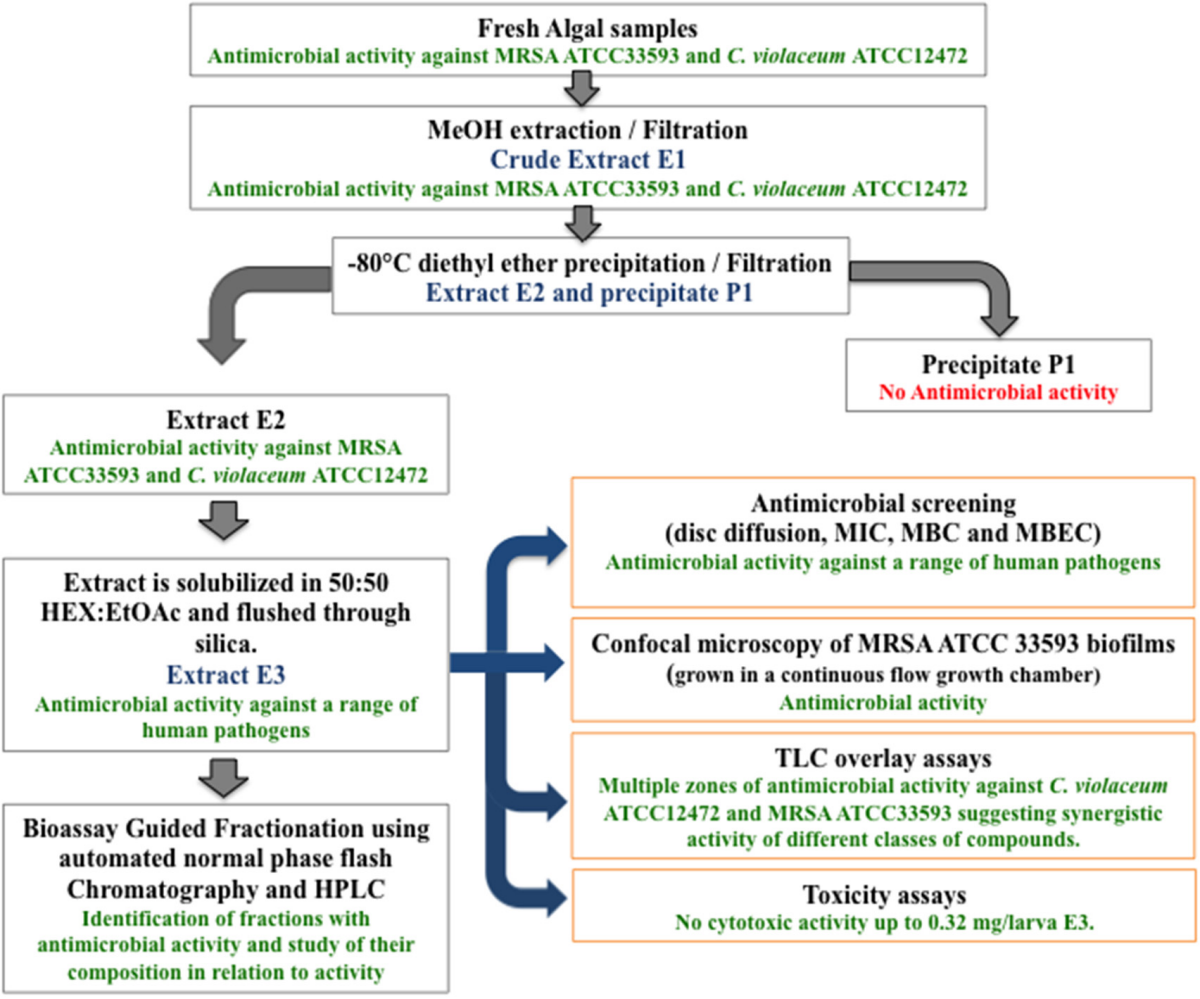
Work flow showing the different steps involved in the extraction and initial bioassay-guided fractionation of *H. siliquosa.* Fresh algal samples were extracted with MeOH yielding crude methanolic extract E1. Extract E1 was de-proteinized yielding extract E2 and precipitate P1. Extract E2 was eluted through silica using hexane:ethyl acetate yielding extract E3 which was further screened for antimicrobial and antibiofilm activity against a panel of clinically-relevant human pathogens. Extract E3 was further fractionated using flash chromatography and active fractions analyzed using HPLC prior to future purification and structural elucidation.

The workflow showing the different steps involved in the extraction and initial bioassay-guided fractionation of *H. siliquosa* yielding 10 active fractions from which to perform the isolation and characterization of novel antibiofilm compounds is shown in Figure 1.

### 2.1. MIC, MBC and MBEC Values of Extract E3

The MIC, MBC and MBEC values for extract E3 against a focused panel of human pathogens was determined (Table 1). The results confirmed the susceptibility results observed using the disc diffusion assay. Planktonic cultures of the Gram positive pathogens *S. aureus* ATCC 29213, *S. aureus* NCTC 12981, *S. aureus* MRSA ATCC 33593, *S. aureus* MRSA 10442 and *S. aureus* MRSA ATCC 43300 proved susceptible to extract E3 with MIC and MBC values ranging from 0.1562 to 0.3125 mg/mL. Biofilms of *S. aureus* MRSA 33593 and *S. aureus* MRSA 10442 were found to be susceptible to extract E3 with MBEC values of 1.25 mg/mL and 5 mg/mL respectively. Planktonic cultures of *S. epidermidis* 35982, *S. epidermidis* 13360, *S. epidermidis* 12228 and *S. epidermidis* MRSE 11964 were also susceptible to the extract with MIC values ranging from 0.1562 mg/mL to 0.625 mg/mL and MBC values ranging from 0.3125 mg/mL to 1.25 mg/mL however the mature biofilm of all three strains proved resistant to the extract with no MBEC values observed. Planktonic cultures of *Staphylococcus haemolyticus* NCTC 11042 and *Staphylococcus hominis* NCTC 11320 were also susceptible to the extract with MIC values ranging from 0.1562 mg/mL to 0.625 mg/mL and MBC values ranging from 0.3125 mg/mL to 1.25 mg/mL whereas biofilm cultures proved resistant, with no MBEC values observed up to and including the highest concentrations of extract tested (5 mg/mL). Planktonic cultures of *Streptococcus pyogenes* NCTC8306, *Streptococcus agalactiae* NCTC 8542 and *Streptococcus pneumonia* NCTC7465 proved particularly susceptible to the extract with MIC values ranging from 0.0391 mg/mL to 0.1562 mg/mL and MBC values ranging from 0.0391 mg/mL to 0.1562 mg/mL whereas biofilm cultures of *Streptococcus pyogenes* NCTC8306 proved resistant, with no MBEC values observed (>5 mg/mL). Planktonic cultures of *Streptococcus sanguinis* NCTC 7863 were resistant to extract E3 at the highest concentration tested (5 mg/mL). Planktonic cultures of *Enterococcus fecalis* 779 were found to be susceptible to extract E3 with and MIC and MBC value of 0.3125 mg/mL and 0.625 mg/mL however complete eradication of mature biofilms was not achieved (MBEC > 5 mg/mL). The Gram negative pathogens *Proteus mirabilis* ATCC 7002, *P. aeruginosa* PAO1, *P. aeruginosa* NCTC 12903, *Escherichia coli* NCTC 12241, and *E. coli* ATCC 11303 proved less susceptible to extract E3. However, *Stenotrophomonas maltophilia* NCTC 10257 (MIC = 0.3125 mg/mL, MBC = 0.625 mg/mL, MBEC = 5 mg/mL) and *C. violaceum* ATCC 12472 (MIC = 0.1562 mg/mL, MBC = 0.3125 mg/mL) were both found to be susceptible to the extract. The yeast *Candida albicans* failed to display susceptibility to the extract E3 using the disc diffusion assay and the effect of the extract on this pathogen was not studied further.

**Table 1:** Antimicrobial and antibiofilm activity of *H. siliquosa* extract E3.

Pathogenic Strain	MIC (mg/mL)	MBC (mg/mL)	MBEC (mg/mL)
*S. aureus* ATCC 29213	0.3125	0.3125	NoA
*S. aureus* NCTC 12981 (ATCC 25923)	0.1562	0.3125	NT
*S. aureus* MRSA ATCC 33593	0.1562	0.1562	1.25
*S. aureus* MRSA NCTC 10442	0.1562	0.3125	5
*S. aureus* MRSA ATCC 43300	0.3125	0.3125	NoA
*S. epidermidis* ATCC 35982	0.1562	0.3125	NT
*S. epidermidis* NCTC 13360 (ATCC 12228)	0.1562	0.3125	NoA
*S. epidermidis* MRSE NCTC 11964	0.625	1.25	NoA
*S. haemolyticus* NCTC 11042	0.1562	0.3125	NoA
*S. hominis* NCTC 11320	0.3125	0.3125	NoA
*S. pyogenes* NCTC 8306 (ATCC 12204)	0.0391	0.0391	NoA
*S. agalactiae* NCTC 8542	0.1562	0.1562	NT
*S. pneumoniae* NCTC 7465	0.0391	0.0781	NT
*S. sanguinis* NCTC 7863	NoA	NoA	NoA
*E. faecalis* ATCC 779	0.3125	0.625	NoA
*P. mirabilis* ATCC 7002	1.25	NoA	NoA
*P. aeruginosa* PAO1	2.5	5	NoA
*P. aeruginosa* NCTC 12903 (ATCC 27853)	2.5	5	NT
*S. maltophilia* NCTC 10257 (ATCC 13637)	0.3125	0.625	NoA
*E. coli* NCTC 12241	5	5	NT
*C. violaceum* ATCC 12472	0.1562	0.3125	NoA
*C. albicans*	NoA	NoA	NoA

Minimum inhibitory concentration (MIC), Minimum bactericidal concentration (MBC) and Minimum biofilm eradication concentration (MBEC) of *H. siliquosa* extract E3. Values are expressed in mg/mL. NoA = no activity observed up to and including the highest concentration of extract tested (5 mg/mL). NT = not tested.

**Figure 2:**
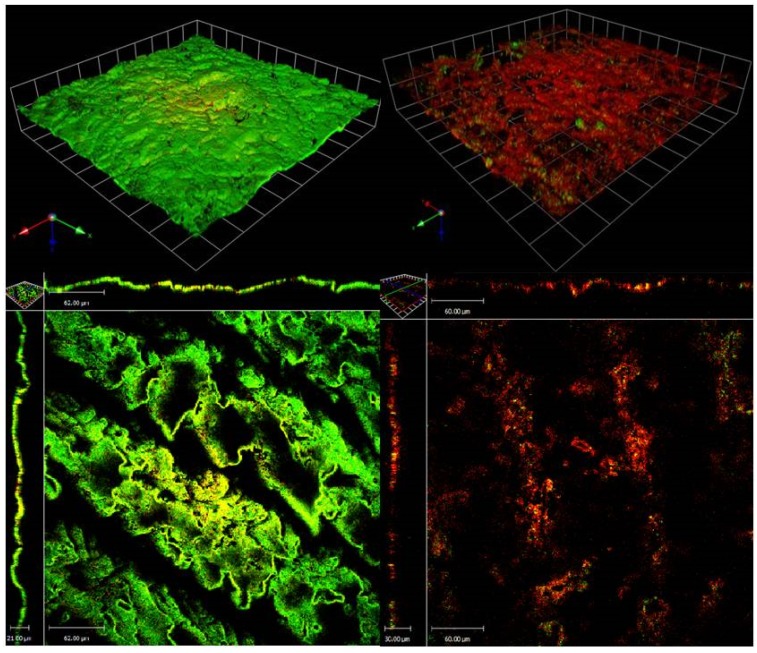
LIVE/DEAD staining and CLSM (60×) of (left) untreated 72 h *S. aureus* (MRSA) ATCC 33593 biofilms and (right) *S. aureus* ATCC 33593 72 h biofilms following 24 h challenge with *H. siliquosa* extract E3 (1.25 mg/mL). Treated MRSA ATCC 33593 biofilms appeared mostly dead (red) confirming the antimicrobial activity seen during screening. The X–Y projections of the treated biofilm suggest a good penetration of the antimicrobial compounds within the exopolymeric matrix with a consistent antimicrobial activity throughout all layers of the biofilm.

### 2.2. CLSM of S. aureus (MRSA) ATCC 33593 Biofilms Challenged with H. siliquosa Extract E3

Following 24 h of growth, control MRSA ATCC33593 biofilms appeared viable, with most of the biofilm cells staining green. Untreated 24 h control biofilms were found to be approximately 7–10 µm in thickness. 24 h MRSA ATCC 33593 biofilms challenged with *H. siliquosa* extract E3 displayed extensive cell death mostly staining red, confirming the antibiotic nature of the bioactive compounds involved (Figure 2). X–Y analysis of the biofilms suggested an effective penetration of the antimicrobials within the biofilms’s exopolymeric matrix, with antimicrobial activity involving all layers of the biofilm, including the lower, typically less metabolically active ones. The extract appeared to affect the integrity of the biomass causing the dispersal of large portions of the biofilm.

### 2.3. Extract Toxicity Screen Using the Galleria Mellonella Model

The *G. mellonella* wax moth larvae model provides a quick, economical, and reliable evaluation of the toxicity of new antimicrobial agents *in vivo* prior to testing using more expensive mammalian models [64]. Extract E3 failed to display toxicity against *G. mellonella* larvae up to and including the highest concentration of extract tested (20 μL of 16 mg/mL working solution) over a period of six days with 100% survival. Controls containing 8% w/v Tween 80 prepared in PBS did not highlight any activity due to the presence of Tween 80 or PBS.

### 2.4. Normal Phase Flash Chromatography/Bioassay Guided Fractionation

**Figure 3:**
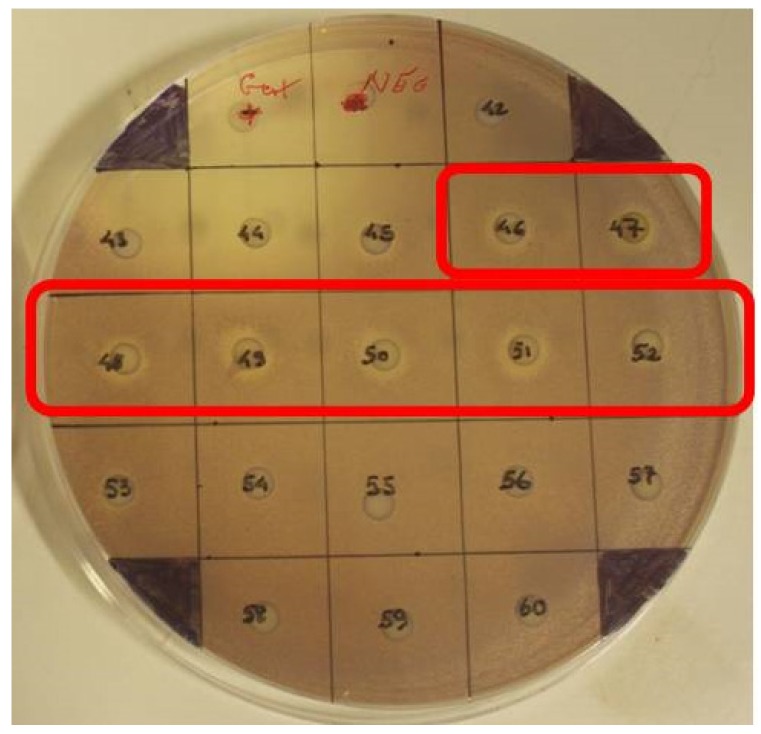
Disc diffusion assay against *C. violaceum* ATCC 12472 of fractions obtained using Flash Chromatography. Antimicrobial activity was detected in fractions 35–36 (not shown) and 46–52.

Extract E3, obtained through Hexane:ethyl acetate elution of extract E2, was fractionated using normal phase automated Flash chromatography into 98 fractions. Each of the 98 fractions was then screened for antimicrobial activity using the disc diffusion assay and the TLC overlay assay using both *C. violaceum* ATCC12472 and *S. aureus* MRSA ATCC 33593. Rf values for UV-visible compounds were calculated and related to compounds displaying activity in the TLC overlay and disc diffusion assays on *C. violaceum* and MRSA 33593. Clear antimicrobial activity against both test strains was present in fractions 35 and 36 and fractions 43–53 (strongest between 46 and 52) (Figure 3).

### 2.5. HPLC Analysis of Fractions

**Figure 4:**
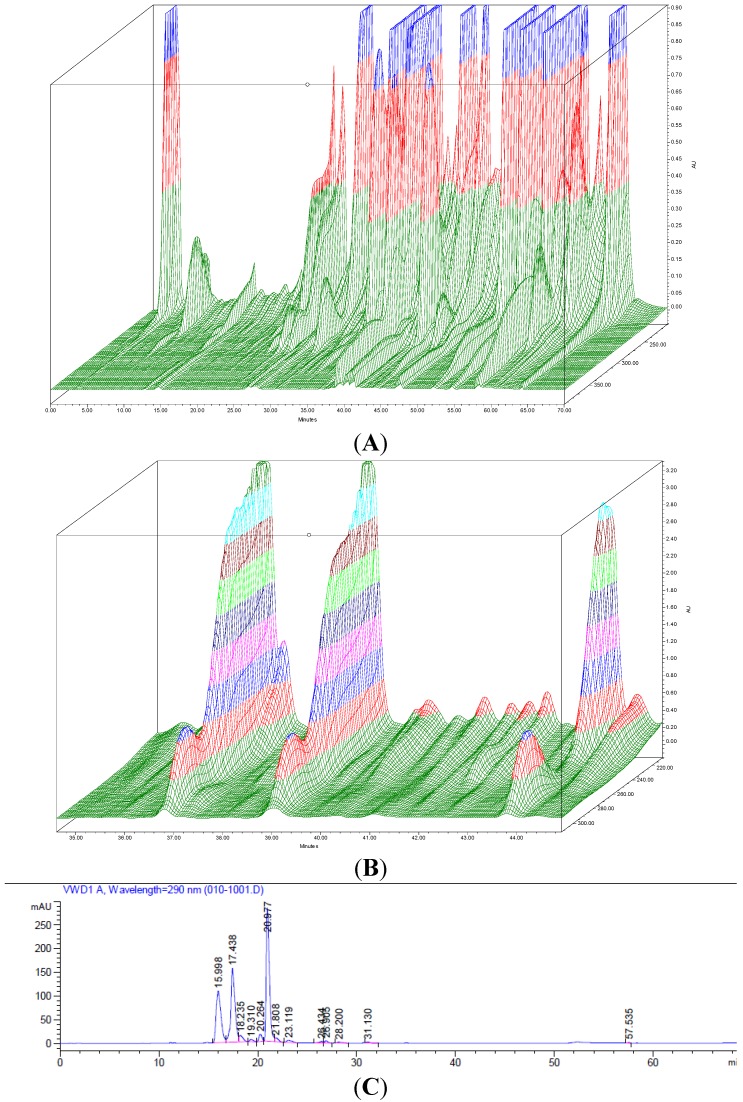
(**A**) 3-dimensional absorbance scan (200–400 nm) using the Photo Diode Array detector (PDA) of extract E3 highlighting the chemical complexity of the extract; (**B**) Main portion of the 3-dimensional absorbance scan of active fraction 48, obtained following bioassay-guided fractionation using automated flash chromatography, showing the presence of 3 main compounds considered to represent the antimicrobial activity identified in the study, each with absorbance peaks at approximately 220 and 290 nm suggesting a similar class of compounds; (**C**) HPLC chromatogram (290 nm) of active fraction 47 displaying the elution pattern of the three compounds (green, white and red arrows) putatively responsible for the antimicrobial activity detected in fractions obtained previously through normal phase fractionation and tested using disc diffusion and TLC overlays.

The composition of the methanolic extracts E1 and E2 and extract E3 (obtained through Hexane:ethyl acetate elution of extract E2) prior to fractionation and of the 98 fractions obtained using normal phase flash chromatography of extract E3, were analyzed using reverse phase HPLC equipped with a photodiode array detector (PDA) set to analyze wavelengths of 200–400 nm. The analysis of extracts E1, E2 and E3 confirmed the removal of putatively non-active constituents present in extract E1 following diethyl ether precipitation/filtration (extract E2) and silica binding (extract E3). As expected, the 3-dimensional chromatogram of extract E3 (Figure 4A) displayed a lower chemical complexity than the chromatograms obtained for the initial crude extract E1 and extract E2 (data not shown). The HPLC analysis of the 98 fractions obtained performing automated flash chromatography confirmed the successful fractionation of extract E3 with each fraction containing between 2 and 7 compounds. The composition and elution pattern of the compounds present in the 10 active fractions obtained using normal flash chromatography and analyzed using the PDA suggests a correlation between the antimicrobial activity observed against MRSA 33593 and the presence of 3 peaks (compounds) which can be seen displaying similar absorption, in the most active fraction, fraction 48 (Figure 4B,C).

### 2.6. Screening Fresh H. siliquosa Fronds for Antimicrobial and QSI Activity

**Figure 5:**
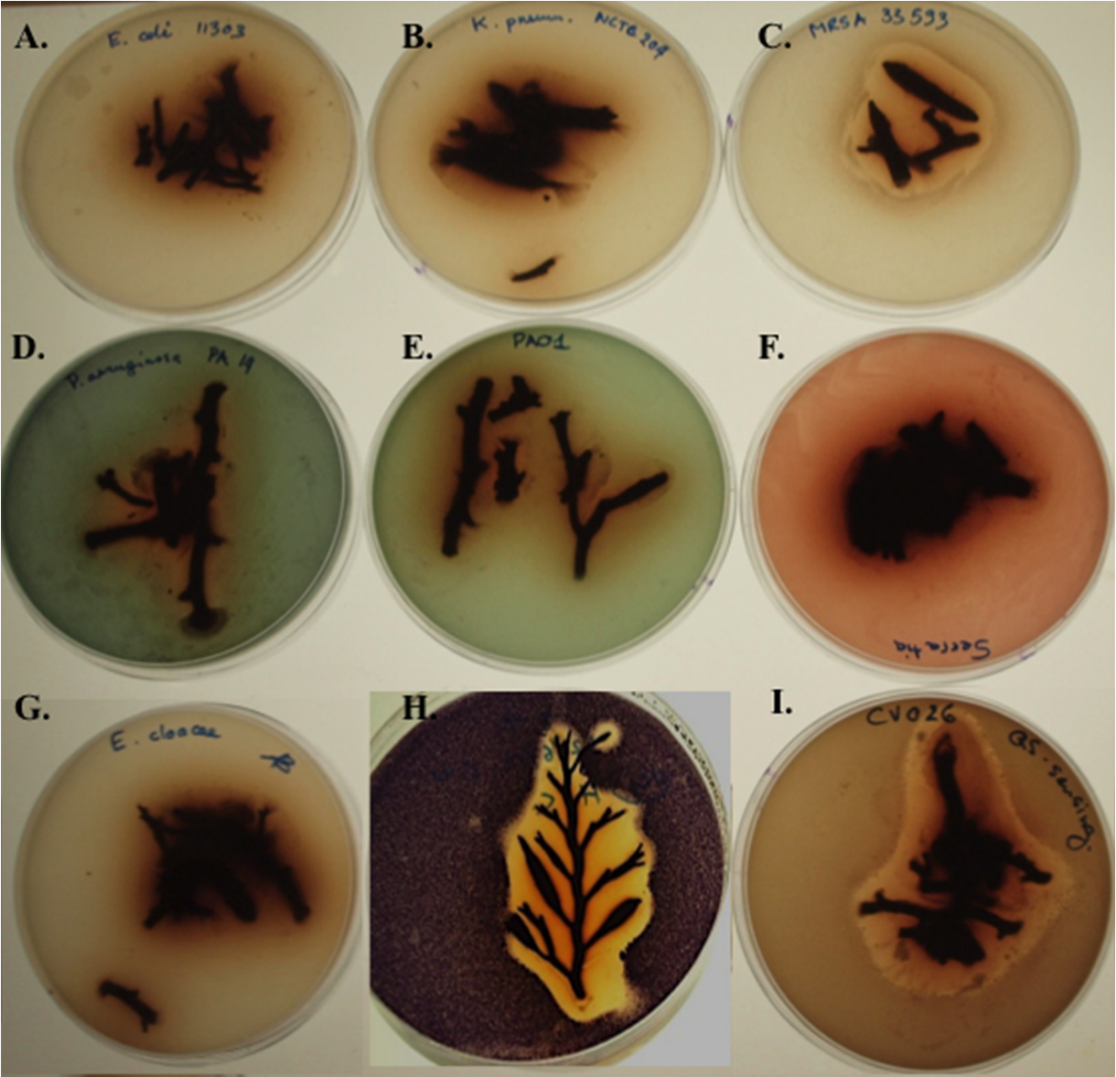
Screening fresh *H. siliquosa* fronds for antimicrobial, QSI and QS activity using the overlay method. (**A**) Very weak antimicrobial activity (2 mm inhibition zone) against *E. coli* ATCC 11303; (**B**) No antimicrobial activity detected against *K. pneumonia* NCTC 204; (**C**) Pronounced antimicrobial activity of *H. siliquosa* against *S. aureus* (MRSA) ATCC 33595; (**D**,**E**) Weak antimicrobial activity of *H. siliquosa* against *P. aeruginosa* PAO1 and PA14; (**F**) No antimicrobial nor QSI activity detected against *Serratia* sp. ATCC39006; (**G**) No antimicrobial activity against *E. cloacae*. (**H**,**I**) Pronounced antimicrobial activity of *H. siliquosa* against QSI reporter strain *C. violaceum* ATCC 12472 and QS reporter strain *C. violaceum* CV026.

The overlay method [65] was used to screen fresh algal fronds for antimicrobial activity against *E. coli* ATCC 11303, *P. aeruginosa* PAO1, *P. aeruginosa* PA14, *E. cloacae, E. faecium* DSM 25390, *S. aureus* MRSA ATCC 33593, and *K. pneumonia* 204. Marked antimicrobial activity was observed against *S. aureus* MRSA ATCC 33593 and the two QS-reporter strains *C. violaceum* ATCC 12472 and *C. violaceum* CV026 and weak antimicrobial activity was observed against *P. aeruginosa* PAO1 and PA14 (Figure 5). A very weak antimicrobial activity was detected against *E. coli* ATCC 11303 consisting in an inhibition zone of approximately 2 mm surrounding the fresh algal sample. The pronounced antimicrobial activity displayed by fresh *H. siliquosa* fronds against reporter strains *C. violaceum* ATCC 12472 and CV026 prevented the detection of QSI or QS compounds using these two reporter strains. The overlay of fresh *H. siliquosa* fronds using QSI reporter *Serratia* sp. ATCC 39006 failed to detect relevant QSI or antimicrobial activity against this strain.

### 2.7. Disc Diffusion Assays Using Halidrys siliquosa Extracts

**Figure 6:**
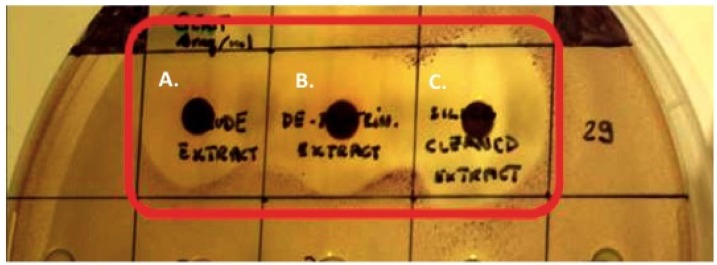
Antimicrobial activity of *Halidrys siliquosa* extracts. Disc diffusion assay on *C. violaceum* ATCC12472 of 100 µL of (**A**) crude methanolic extract E1 (4 mg/mL) (left), (**B**) extract E2 (4 mg/mL) (center) and (**C**) extract E3 (4 mg/mL) (right).

The crude methanolic extract (E1), the diethyl ether treated, de-proteinized extract (E2), the precipitate P1 and the extract (E3) of *H. siliquosa* were screened for antimicrobial activity using the disc diffusion assay. Antimicrobial susceptibility discs loaded with 100 µL of extracts E1, E2 and E3 at 4 mg/mL all displayed strong antimicrobial activity against *S. aureus* (MRSA) ATCC 33593 (with diameters of inhibition of E1 = 10 mm, E2 = 12 mm, E3 = 13.5 mm) and *C. violaceum* ATCC 12472 (Figure 6) (E1 = 10 mm, E2 = 11 mm, E3 = 12 mm). P1 failed to display significant antimicrobial activity.

Disc diffusion assays were used to screen extract E3 for antimicrobial activity against a panel of clinically relevant human pathogens. *H. siliquosa* extract E3 was found to be active against the Gram positive pathogens of the genus *Staphylococcus, Streptococcus,* and *Enterococcus* and the Gram negative pathogens *C. violaceum*, *P. mirabilis*, and *S. maltophilia.* *P.* *aeruginosa* PAO1 was not found to be susceptible to the extract. The results of the disc diffusion assays are summarized in Table 2.

**Table 2:** Antimicrobial activity of *Halidrys siliquosa* extract E3.

Pathogenic Strain	E5	CIP1	P1	TE10	CN10	Extract E3 (4 mg/mL)
*S. aureus* ATCC 29213	(−)	19 mm	9 mm	22 mm	16 mm	11 mm
*S. aureus* NCTC 12981 (ATCC 25923)	19 mm	(−)	11 mm	(−)	16 mm	11.5 mm
*S. aureus* MRSA ATCC 33593	(−)	19 mm	(−)	(−)	8 mm	13.5 mm
*S. aureus* MRSA NCTC 10442	(−)	18 mm	(−)	(−)	16 mm	13 mm
*S. aureus* MRSA ATCC 43300	(−)	16 mm	8 mm	18 mm	9 mm	10.5 mm
*S. epidermidis* ATCC 35982	(−)	26 mm	(−)	22 mm	14 mm	13.5 mm
*S. epidermidis* NCTC 13360 (ATCC 12228)	21 mm	22 mm	(−)	(−)	20 mm	12 mm
*S. epidermidis* MRSE NCTC 11964	11 mm	(−)	(−)	(−)	(−)	7 mm
*S. haemolyticus* NCTC 11042	18 mm	20 mm	(−)	(−)	16 mm	13.5 mm
*S. hominis* NCTC 11320	27 mm	25 mm	26 mm	(−)	26 mm	10 mm
*S. pyogenes* NCTC 8306 (ATCC 12204)	(−)	26 mm	(−)	20 mm	15 mm	15 mm
*S.* *agalactiae* NCTC 8542	20 mm	15 mm	11 mm	21 mm	17 mm	13 mm
*P. mirabilis* ATCC 7002	(−)	26 mm	(−)	(−)	16 mm	6 mm
*P. aeruginosa* PAO1	(−)	19 mm	(−)	(−)	18 mm	0 mm
*S. maltophilia* NCTC 10257 (ATCC 13637)	(−)	30 mm	(−)	16 mm	20 mm	10 mm
*E. coli NCTC 12241*	18 mm	14 mm	11 mm	27 mm	19 mm	10 mm
*C. violaceum* ATCC 12472	20 mm	32 mm	(−)	25 mm	20 mm	12 mm
*C. albicans*	n.a.	n.a.	n.a.	n.a.	n.a.	0 mm

Antimicrobial activity of *Halidrys siliquosa* extract E3. Zones of inhibition (mm) using the disc diffusion assays. Control antibiotics: Erythromycin 5 µg (E5), Penicillin 1 Unit (P1), Tetracycline 10 µg (TE10), Ciprofloxacin 5 µg (CIP1) and Gentamycin 10 µg (CN10). Disc diffusion using 100 µL of MeOH extract at 4 mg/mL. (−) = no. activity detected, n.a. = not tested (yeast).

### 2.8. TLC Overlay Assays

The extracts E1, E2 and E3 were resolubilized in MeOH and developed on normal phase TLC plates using MeOH:Hex 50:50 as a mobile phase. The plates were screened for antimicrobial activity by overlaying with soft LB agar inoculated with either *C. violaceum* ATCC 12472 or *S. aureus* (MRSA) ATCC 33593. Two distinct bands of antimicrobial activity were detected against *S. aureus* (MRSA) ATCC 33593 (results not shown). Three distinct bands of antimicrobial activity were detected against *C. violaceum* ATCC 12472 (results not shown). The two distinct bands of antimicrobial activity observed against MRSA ATCC 33593 coincided (same Rf value) with two of the three bands of antimicrobial activity observed against *C. violaceum* ATCC 12472 suggesting the same 2 compound(s) or groups of compounds contribute to the cumulative antimicrobial activity observed against these two test strains. The results also suggest the presence of two or more classes of antimicrobial compounds with substantially different molecular structures resulting in differing polarities and migratory speed in the TLC system used. We can thus assume the final antimicrobial activity exhibited by the crude extract using the disc diffusion assay or the MIC assay against MRSA ATCC 33593 is the result of the additive or synergistic activity of 2 distinct groups of compounds whereas the activity observed against *C. violaceum* ATCC 12472 is the result of the additive or synergistic activity of 3 distinct groups of compounds.

## 3. Discussion

According to the estimates of the Centers for Disease Control (CDC) and the National Institute of Health (NIH), 65%–80% [66,67] of all bacterial infections worldwide are associated with biofilms [24,68]. Such infections are typically characterized by an inherent resistance to antibiotics, an extraordinary capacity to evade the host immune system [37] and by an increased rate of horizontal genetic transfer leading to the acquisition and spread of antibiotic resistance and multi-resistance. The occurrence of biofilm-mediated infections, especially as a result of the medical use of implantable devices and catheters, is on the rise [69,70] and the identification of novel compounds with the capacity to inhibit bacterial colonization and biofilm formation is of crucial importance. The production of bioactives and in particular antimicrobials synthesized by the brown macroalgae is well documented in the literature. In this work, the marine brown alga *Halidrys siliquosa* was screened for the production of antimicrobial and antibiofilm compounds against a panel of clinically relevant human pathogens commonly associated with biofilm-related infections such as Cystic Fibrosis (CF) and infections associated with the use of indwelling medical devices such as urinary catheters [71,72] or intravenous catheterization in the nosocomial environment [73,74]. A simple protocol yielding an easily replicable organic extract designated E3 with a defined composition suitable for the purification of antibiofilm compounds produced by this alga was developed (Figure 1).

Disc diffusion assays using the organic extract E3 revealed a broad-spectrum antimicrobial activity against Gram positive pathogens of the genus *Staphylococcus*, and *Streptococcus*. The Gram negative pathogen *P. mirabilis* ATCC7002 was found to be less susceptible to the extract than many of the susceptible Gram positive test strains. The Gram negative pathogen *S. maltophilia* NCTC10257 was also found to be susceptible to the extract, with an inhibition zone similar to that observed for many of the Gram positive pathogens found to be susceptible. *S. maltophilia* is considered an emerging opportunistic pathogen most frequently associated with pulmonary infections with a significant fatality/case ratio [75,76]. On the contrary, the Gram negative *P. aeruginosa* PAO1 and the yeast *Candida albicans* were found to be insensitive to the extract E3.

MIC, MBC and MBEC values of extract E3 confirmed the susceptibility of several test strains. Importantly, biofilms of *S. aureus* (MRSA) 33593 and *S. aureus* (MRSA) NCTC10442 were found to be susceptible to the extract with MBEC values of 1.25 mg/mL and 5 mg/mL respectively. The increased rate of infections caused by methicillin-resistant *S. aureus* (MRSA) and the treatment-limiting toxicities of many current antibiotics highlight the growing need for novel drugs [43]. LIVE/DEAD staining of *S. aureus* ATCC 33593 biofilms following 24 h challenge with algal extract viewed using CLSM showed extensive cell death in mature biofilms treated with extract E3 confirming the antibiotic nature of the bioactive compounds involved. Although planktonically grown cultures of *E. coli* (NCTC12241), the single largest cause of catheter-associated UTIs (CAUTIs) [71], were found to be susceptible to the algal extract (MIC = MBC = 5 mg/mL), the biofilms of this pathogens remained unaffected across the range of concentrations tested (up to and including 5 mg/mL) with no MBEC value observed. The extract was also found to be active against plaktonic cultures of *E. faecalis* ATCC 779 (MIC = 0.3125 mg/mL, MBC = 0.625 mg/mL) however biofilms of this pathogens exhibited no susceptibility.

The agar overlay method was used to screen fresh *H. siliquosa* algal fronds for antimicrobial activity against a small panel of human pathogens. Significant antimicrobial activity was observed against *S. aureus* MRSA ATCC33593. A weak antimicrobial activity was observed against *P. aeruginosa* PAO1, PA14 and *Escherichia coli* ATCC 11303. No antimicrobial activity was detected against *K. pneumonia* NCTC 204, *E. cloacae* or *E. faecium* DSM 25390. Although the overlay protocol requires relatively little sample preparation and is relatively high-throughput, care must be taken in interpreting the results as the inhibitions observed could be attributable to the production of bioactives by microbial epiphytes, the presence of contaminants or residual sodium chloride. Moreover, the effects of seasonal and geographical variation on bioactive production are well documented and in 1976, Hornsey and Hide examined the relationship between the production of bioactives and seasons reporting a spring time peak of antimicrobial activity for *H. siliquosa* [22].

Disc diffusion assays on *S. aureus* (MRSA) ATCC 33593 and *C. violaceum* ATCC 12472 using extracts E2 and P1 confirmed the presence of antimicrobial activity in E2 but not in P1 suggesting the bioactive(s) responsible for the antimicrobial activity were effectively extracted using methanol and are not proteinaceous in nature. All three extracts (E1, E2 and E3) displayed strong antimicrobial activity against *S. aureus* (MRSA) ATCC 33593 and *C. violaceum* ATCC 12472 with the antimicrobial activity increasing from E1 to E3. This gradual increase in antimicrobial activity E1–E3 can be explained in part by the gradual removal of non-active constituents and the consequential rise in the relative concentration of the bioactives responsible for the antibiotic activity observed.

Quorum sensing inhibition represents a novel approach to attenuate bacterial virulence and limit the emergence of pathogenic traits, causing bacteria to fail to adapt to the host environment and establish an infection [62,77]. Quorum sensing inhibitors from marine algae have been reported previously, most notably halogenated furanones from the red alga *Delissea pulchra* [10,78,79]. Accordingly, *H. siliquosa* was screened for QS and QSI activity using Gram negative *N-*acyl-homoserine lactone (AHL)-based reporters. Fresh algal fronds were overlaid with QS reporter strain *C. violaceum* CV026 and QSI reporters *C. violaceum* ATCC 12472 and *Serratia* sp. ATCC 39006. The AHL QS system in *Serratia* sp. ATCC39006 relies on *smaI* and *smaR* (secondary metabolite activator) to regulate the synthesis of two AHL signalling molecules, *N*-butanoyl-l-homoserine lactone (C4-HSL) and *N*-hexanoyl-l-homoserine lactone (C6-HSL). At high cell densities, these two AHLs inhibit the DNA binding activity of SmaR (de-repression), resulting in the production of PigQ, PigR and Rap, and the activation of prodigiosin biosynthesis [80]. The Gram negative human pathogen *P. aeruginosa* utilizes an analogous locus, the *rhl* system, where *rhlI* directs the synthesis of *N*-(butanoyl)-l-homoserine lactone (C4-HSL), which interacts with the cognate regulator *rhlR* and activates target gene promoters. In *P. aeruginosa*, the RhlR-C4-HSL complex has been found to regulate the expression of *rhlAB*, required for rhamnolipid production, *lasB*, *aprA*, the stationary-phase sigma factor, RpoS, and the production of secondary metabolites such as pyocyanin and cyanide [81,82].

Pigment production by reporter strain *Serratia* sp. ATCC 39006 was not affected in the proximity of the fresh fronds suggesting the absence of QSI compounds capable of interfering with the C4-HSL-based QS-pathway. The strong antimicrobial activity displayed by the organic algal extract against reporters *C. violaceum* ATCC 12472 and *C. violaceum* CV026 prevented the detection of QSI or QS inducing compounds.

The TLC overlay assay was conducted both to study the composition of the organic extracts E1, E2 and E3 and of the 98 fractions obtained using flash chromatography, and to identify the compound(s) responsible for the antimicrobial activity observed. The TLC overlay assay is a convenient method which aids in determining whether an antimicrobial activity is attributable to the presence of a single compound or multiple compounds. Agar overlays of TLC plates of extract E3 highlighted the presence of 2 distinct groups of antimicrobial compounds active against *S. aureus* (MRSA) ATCC33593 and 3 distinct groups of compounds active against *C. violaceum* ATCC12472. Therefore, the antimicrobial activity observed when performing the disc-diffusion assay against the test pathogens used in the study is likely to be the result of an additive effect of multiple bioactives. Moreover, the fact that the two bands of inhibition observed against *S. aureus* MRSA ATCC33593 have the same Rf values as the ones observed against *C. violaceum* ATCC12472 suggests that the same bioactives could be in part responsible for the bioactivities observed against these two test strains. Whilst *C. violaceum* does not represent a significant target pathogen in its own right, it possesses an antimicrobial susceptibility profile comparable to clinically relevant pathogens such as the ones displayed by the *S. aureus* and MRSA strains used in this study with the benefit of providing a clear zone of clearance against an otherwise pigmented lawn of bacteria. Although the TLC overlay assay allows the separation and identification of different activities within an extract, its resolution is limited and dependant on the utilization of an *ad hoc* mobile phase allowing the effective separation of bioactive compounds with comparable polarity. Moreover, the TLC system used allowed the visualization of compounds that absorb in the visible region of the light spectrum and at 254 or 265 nm limiting the portion of compounds that could be visualized. For these reasons, further fractionation was performed using automated Flash chromatography and fractions analyzed using an HPLC system equipped a PDA detector allowing a deeper and more accurate analysis of the composition of the extracts or fractions being analyzed. Based on the composition analysis of the active fractions and the elution pattern of the compounds present in the 98 fractions obtained through normal phase fractionation and monitored using HPLC at 290 nm and 220 nm, the antimicrobial activity detected in fractions 42–52 using the disc diffusion and TLC overlay assays can be correlated to the presence of 3 peaks which can be seen in the crude extract with retention times of 15.7 min, 17.2 min and 20.8 min (Figure 4B,C). The extraction and bioassay-guided fractionation method developed provides a simple replicable protocol yielding 10 active fractions from which to perform purification and structural elucidation of clinically-relevant antibiofilm compounds produced by *H. siliquosa*. Structural elucidation of the three bioactive compounds responsible for the antimicrobial and antibiofilm activity observed is currently underway.

The wax moth *G. mellonella* provides a non-mammalian model for evaluating the toxicity of novel antimicrobial agents *in vivo* [83]. Toxicity studies using the *G. mellonella* model showed no toxicity of extract E3 up to 0.32 mg/larvae over a period of 6 days with 100% survival of larvae treated with 20 µL of 16 mg/mL E3. Future work will attempt to assess the antimicrobial efficacy *in vivo* of *H. siliquosa* extract using this model. The variety of antimicrobial activities observed using methods such as the TLC overlay coupled with the absence of toxicity against *G. mellonella* suggests this alga remains an ideal subject for future studies involving the identification, purification and structural elucidation of marine-derived, medically relevant natural products.

## 4. Experimental Section

### 4.1. Bacterial Strains Used in This Study

Bacteria used in this study were *Staphylococcus aureus* (MRSA) ATCC 10442, *S. aureus* (MRSA) ATCC 33593, *S. aureus* (MRSA) ATCC 43300, *S. aureus* ATCC 12981, *S. aureus* ATCC 29213, *Staphylococcus epidermidis* MRSE ATCC 11969, *S. epidermidis* ATCC 13360, *S. epidermidis* ATCC 35982, *S. epidermidis* ATCC 12228, *Staphylococcus haemolyticus* ATCC 11042, *Staphylococcus hominis* ATCC 11320, *Streptococcus pneumoniae* NCTC 7465, Streptococcus sanguinis ATCC 7863, *Streptococcus pyogenes* ATCC 8306, *Streptococcus agalactiae* NCTC 8542 AB, *Enterococcus fecalis* ATCC 779, *Stenotrophomonas maltophilia* ATCC 10257, *Proteus mirabilis* ATCC 7002, *Pseudomonas aeruginosa* (PAO1), *P. aeruginosa* (PA14), *P. aeruginosa* ATCC12903, *Escherichia coli* ATCC 11303, *E. coli* ATCC 12241, *E. coli* ATCC 8196, *E. cloacae*, *Enterococcus faecium* DSMZ 25390, *Klebsiella pneumonia* NCTC 204 and the yeasts *Candida albicans* and *C. tropicalis* NCTC 7393. All pathogenic test strains were cultured in Luria Bertani (LB) broth at 37 °C with shaking at 100 rpm unless otherwise specified.

*C. violaceum* ATCC 12472 (cultured in LB broth at 37 °C) and *Serratia* sp. ATCC 39006 (cultured in LB broth at 28 °C) were used to screen for the production of QSIs. *C. violaceum* CV026 (cultured in LB broth (kanamycin 25 µg/mL) at 28 °C) was used to screen for the production of AHL-based quorum sensing (QS) inducers.

All strains used in this study were stored in cryovials containing overnight cultures in LB broth supplemented with 15% glycerol at −80 °C.

### 4.2. Galleria Mellonella Larvae Used in the in Vivo Toxicity Study

Sixth-instar *G. mellonella* larvae were obtained commercially from livefoodsdirect.co.uk and stored at 15 °C prior to use. Dead larvae and those with dark spots or showing signs of melanisation were discarded.

### 4.3. Chemicals and Reagents

Chemicals and solvents were purchased from Sigma Aldrich (Poole, Dorset, UK) and VWR international (Lutterworth, UK). All reagents and solvents were of the highest purity and were used without further purification.

### 4.4. Sample Collection

A quantity of 2.25 kg (wet weight) of fresh specimens of *Halidrys siliquosa* was collected by SCUBA diving at several locations along the northern coast of the island of Ireland. A small portion of fresh alga was used to screen fresh algal fronds. The remainder was stored at −80 °C.

### 4.5. Screening Fresh H. siliquosa for Antimicrobial and QSI Activity

Fresh fronds of *H. siliquosa* were screened for antimicrobial and QSI activity using the protocol developed by McLean *et al*. [65] with slight modifications; fronds were cut to a suitable size and were rinsed in sterile-filtered seawater (SSW) before being placed onto an LB agar plate and overlaid with 10 mL of LB 0.5% agar containing an overnight culture of *C. violaceum* ATCC12472 (5 μL), *Serratia* sp. ATCC39006 (5 μL), *C. violaceum* CV026 (50 μL) or the different pathogenic test strains (50 μL) to be tested. Following overnight incubation plates were examined for the presence of clear halos indicative of antimicrobial activity, opaque halos surrounding *C. violaceum* ATCC12472 or *Serratia* sp. ATCC39006 indicative of QSI, or violacein production by CV026 in the presence of exogenous autoinducer.

### 4.6. Solvent Extraction of Halidrys siliquosa

Fresh samples of *H. siliquosa* were extracted at room temperature (RT) with HPLC-grade methanol (MeOH). Fresh algal samples (blades and thalli) were cleaned manually removing any visible epiphytes and then washed thoroughly with SSW to remove any remaining debris. Two hundred grams of washed sample was placed in a glass beaker and extracted at room temperature in 400 mL of MeOH, on a horizontal shaker (90 rpm) for 4 h. The first methanolic extract was then collected and replaced with 400 mL of fresh MeOH for an additional 4 h. A total of three extractions were performed on each algal sample. The combined extracts were filtered using Whatman No 1 filter paper (Oxoid, UK) and dried using a Büchi Rotavapor R-210 (Flawil, Switzerland) in a water bath at 30 °C. The resulting dark brown oil was freeze-dried in an Edwards Modulyo benchtop freeze drier (Edwards, UK). Following filtration and freeze-drying, the blades and thalli (200 g wet weight) of *H. siliquosa* yielded 2.865 g of crude extract. The crude methanolic extract was designated (E1).

The crude extract was then re-solubilized in 100 mL of MeOH and 100 mL of ice-cold diethyl ether and kept overnight at −80 °C inducing the precipitation of proteinacous components of the crude extract thereby allowing a first crude partitioning of the compounds originally in the crude extract. Following overnight incubation at −80 °C, the methanolic extract was filtered using a Whatman No 1 filter paper. Overnight precipitation and filtration were repeated twice. The extract was then reduced to dryness under reduced pressure in a rotary evaporator at a temperature not exceeding 30 °C. This secondary methanolic extract was designated (E2). The proteinaceous precipitate was dried under a stream of nitrogen. This extract was designated (P1).

To remove any compounds that would bind irreversibly to silica, the dried extract E2 (2.3 g) was re-solubilized in 5 mL of Hex:EtOAc 50:50 and eluted through a glass column packed with silica gel 60A (Fluorochem, Hadfield, UK) using 1.5 L of Hex:EtOAc 50:50 as the mobile phase. This extract was designated (E3).

Dried extracts E1, E2, E3 and precipitate P1 were stored in glass vials at −20 °C. The strategy used for the extraction and testing of *Halidrys siliquosa* extract is summarized in Figure 1.

### 4.7. Screening for Antimicrobial Activity—Disc Diffusion Assay

The antibiotic susceptibility testing on pathogenic test strains was performed using a modified version of the Kirby Bauer disc-diffusion method [84]. Extracts E1, E2, E3 and precipitate P1 re-solubilized in MeOH to yield solutions of 10 mg/mL. The re-solubilised solutions were pipetted onto sterile paper disks 6 mm in diameter (Whatman, UK) by transferring a maximum of 10 µL volumes at a time to achieve the desired test concentrations. Air-drying was allowed between multiple loadings. Sterile forceps were used to transfer the dried discs in triplicate, onto single LB agar plates. A disc loaded with 100 µL MeOH and allowed to air dry was also included as a solvent control. Control discs of standard antibiotics were used when appropriate: Penicillin 1 Unit (P1), Tetracycline 10 µg (TE10), Erythromycin 5 µg (E5), Gentamycin 10 µg (CN10) and Ciprofloxacin 5 µg (CIP1) (Oxoid Limited, Thermo Scientific, Basingstoke, UK). The diameter of the zone of inhibition (mm) was calculated as the mean of three independent experiments (biological replicates).

The LB agar plates were then overlaid with 10 mL of LB 0.5% agar inoculated with 5 µL of an overnight culture of *C. violaceum* ATCC12474 or *Serratia* sp. ATCC39006 or 50 μL of overnight culture of each pathogenic test strain to be tested. Three replicate plates of each extract were prepared. Plates were incubated overnight at 37 °C or 28 °C for 24 h before being examined for the presence of opaque halos indicating QSI inhibition or clear halos indicating antimicrobial activity.

### 4.8. MIC/MBC Determination

Broth microdilution tests were carried out based on the protocol described in NCCLS guidelines (NCCLS, 2000), with slight modifications. A working solution of each extract to be tested was prepared by dissolving the extract E3 in Tween 80 (8% w/v) and LB broth and sterilised using a 0.22 µm filter. From this stock solution, serial two-fold dilutions in LBB were carried out in 96-well microtitre plates over the concentration range 5–0.0024 mg/mL. Test organisms were grown for 24 h at 37 °C in LB broth. Overnight cultures were used to prepare inocula of approximately 2 × 10^5^ CFU/mL. The microtitre plate for the determination of MIC and MBC was set up including Tween 80 controls. All controls and test concentrations were prepared as a minimum of four replicates and each assay was repeated in triplicate. Microtitre plates were incubated for 24 h at 37 °C in a stationary incubator. Following incubation, the MIC for each extract was determined reading absorbance at 600 nm. The MBCs were derived by transferring 20 µL of the planktonic suspension from the test wells to LB agar plates. Following incubation in a stationary incubator at 37 °C for 24 h plates were examined for 99.9% killing.

### 4.9. Antibiofilm Activity of H. siliquosa Crude Extracts

The MBEC assay was conducted using the *H siliquosa* extract E3 prepared as for the MIC tests, as previously described. A 96-well microtitre plate was inoculated with the test strains as follows: column 1 containing 200 µL of LBB (blank), column 2–12 containing 100 µL of LBB and 100 µL of test inoculum (prepared as previously for MIC determinations at a final density of 2 × 10^5^ cfu/mL). The MBEC assay plates (Innovatech Inc., Edmonton, Canada) were transferred to a gyrorotary incubator (37 °C, 95% relative humidity) for 24 h to allow growth of test biofilms. Negative growth/sterility controls were included in each plate (6 replicates). Planktonic (CFU/mL) and biofilm viable counts (BFU/peg) at 24 h were measured. Following the 24 h growth period, the peg lid of the MBEC assay plate was transferred to rinse plate and each peg gently rinsed three times by immersion in wells containing 300 µL of sterile PBS.

After rinsing, the lid was transferred to a challenge plate containing a range of doubling dilutions of extract E3 over the concentration range of 5–0.0024 mg/mL and an untreated (LB broth) and an LB broth containing Tween 80 (8% w/v) control. Following exposure of the biofilm to the challenge for 24 h the peg lid was removed from the challenge plate and rinsed three times in 300 µL of sterile PBS. After rinsing, four pegs were broken off from each of the test columns and used to determine biofilm viable counts. The lid with the remaining pegs was transferred to a “recovery” plate containing LB broth. Biofilms were sonicated for 5 min and the peg lid discarded. The recovery plate was incubated overnight and visually checked after 24 h for turbidity. In addition, optical density measurements for each plate were recorded at 550 nm, clear wells were taken as evidence of biofilm eradication, and, an MBEC value assigned as the lowest concentration at which no growth was observed after 24 h incubation.

### 4.10. TLC Overlays-Bioassay Guided Fractionation

Normal phase TLC plates (silica gel with fluorescent indicator 254 nm, Sigma-Aldrich, Dorset, UK) were spotted with extracts resolubilized in MeOH and developed using the mobile phase 50:50 MeOH:HEX, allowed to air-dry overnight in a fume hood and then positioned at the bottom of an empty Petri dish. The TLC sheets were then overlaid with 10 mL of 0.5% LBA containing a 1 × 10^8^ cfu/mL inoculum of the test strain. Once set, plates were placed at 37 °C for 24 h before being examined for halos of QSI/antimicrobial activity. The TLC overlay assay was conducted using *C. violaceum* ATCC12472 and *S. aureus* (MRSA) ATCC33593.

### 4.11. Confocal Laser Scanning Microscopy of S. aureus Biofilms Treated with H. siliquosa Crude Extracts

*S. aureus* ATCC 33593 (methicillin and gentamicin resistant) biofilms were grown on polycarbonate coupons (10 mm diameter) in a dual channel continuous flow cell chamber (FC-271-AL, BioSurface Technologies Corp., Bozeman, MT, USA) for 3 days. Fresh LB broth at 37 °C was allowed to flow through the flow cell at a rate of 0.1 mL/min for 3 h to condition the surface of the coupons and facilitate adhesion. The flow was then stopped and 1 mL of mid-log *S. aureus* ATCC 33593 suspension was injected into each of the two chambers of the flow cell and allowed to stand for 1 h to allow initial adhesion of bacterial cells onto the coupons’ surface. Following the adhesion step, the flow of LB broth was turned on at a rate of 0.1 mL/min for 72 h. The LB broth flow was then stopped and the line feeding the growth chamber to be challenged was aseptically connected to a bottle containing 300 mL of LB broth containing filter-sterilised (0.22 μm), *H. siliquosa* extract E3 at 4 mg/mL. The flow was then turned on again at a flow rate of 0.1 mL/min for 24 h. At the end of the challenge period, the two chambers were rinsed with 0.9% NaCl solution through the flow cell (0.4 mL/min) for 10 min before proceeding to staining. Following the rinse step the flow was stopped and biofilms grown on polycarbonate coupons were stained with LIVE/DEAD BacLight Bacterial Viability Kit L13152 (Molecular Probes, Eugene, OR, USA) by injecting 2 mL of staining solution into each chamber and allowing it to stand for 15 min. Light exposure was minimised by covering the flow cell with aluminium foil. After 15 min of staining, 0.9% NaCl solution was allowed to flow through two chambers for 10 min to rinse away any excess die. The flow was then stopped, the flow cell opened, and the coupons mounted on glass slides for examination with confocal laser scanning microscope (Leica TCS SP2 Confocal Microscope, Leica Microsystems, Milton Keynes, UK). Z-stacks of confocal images were rendered into 3D mode using Volocity software (PerkinElmer, Seer Green, UK).

### 4.12. Extract Toxicity Screen Using the Galleria Mellonella Model

From a 50 mg/mL stock solution of E3 containing 8% w/v Tween 80, ten doubling dilutions of each extract were prepared in PBS. Twenty microliters of each concentration (20 µL of the highest concentration equated to 0.32 mg/larva) was inoculated into 10 larvae weighing between 0.2 and 0.3 g, through the base of the last left proleg. Larvae were incubated in Petri dishes containing wood shavings as a source of nutrition at 30 °C for 48 h and examined visually for viability over a period of 6 days. As controls, larvae were treated with sterile PBS alone or with a solution of 8% w/v Tween 80 prepared in PBS.

### 4.13. Bioassay Guided Fractionation of Antibiofilm Bioactives from Halidrys siliquosa

*H. siliquosa* extract E3 was further fractionated using normal phase automated Flash chromatography (Isolera Biotage™). Each of the 98 fractions obtained was then screened for antimicrobial activity using the disc diffusion assay and the TLC overlay assay against *C. violaceum* ATCC12472 and MRSA 33593 prior to analyzing the composition using analytical HPLC.

A quantity equal to 2.5 g of extract was dissolved in 2 mL of Hex/EtOAc 50:50 and loaded onto a Biotage™ SNAP Cartridge KP-Sil 50 g and fractionated using an Isolera Biotage™ automated Flash chromatography system (Biotage, Uppsala, Sweden) using the solvent system A = Hexane, B = EtOAc and the following gradient: V = 0, A = 90%, B = 10%; V = 2100 mL, A = 60%, B = 40%, flow = 25 mL/min. Mode: “collect all” 21 mL per fraction.

The composition of each fraction was monitored by TLC (silica gel with fluorescent indicator 254 nm, Sigma-Aldrich, Dorset, UK) and visualized using a UV lamp 254–365 nm. Retention factor (Rf) values were calculated and related to compounds displaying activity in the TLC overlay and disc diffusion assays on *C. violaceum* and *S. aureus* MRSA 33593.

HPLC analysis was performed using a Waters 2695 HPLC system equipped with a Waters Photodiode Array (PDA) detector 2996 (Waters Limited, Elstree, UK). Analytical analysis of the 98 fractions obtained using normal phase flash chromatography was performed using a Luna^®^ 5 µm C18(2) 100 Å, LC Column 150 × 4.6 mm (Phenomenex^®^, Macclesfield, UK). The analysis was performed at 216 and 254 nm using the solvent system A = H20 + 0.1% formic acid, B = ACN + 0.1% formic acid and the following gradient: t0′ A = 70%, B = 30%; t50′ A = 30%, B = 70%; flow = 2 mL/min.

## 5. Conclusions

The brown macroalga *Halidrys siliquosa* was found to be a rich source of diverse and potentially novel antimicrobial and antibiofilm compounds with clinical relevance. Previous work on this alga had reported antimicrobial activity against a series of opportunistic human pathogens including *S. aureus*, *E. coli*, *Bacillus subtilis*, *Streptococcus pyogenes*, *Proteus morganii* [22] and *Mycobacterium tuberculosis* [19]. In this work, the refined extract E3 obtained with Hexane:ethyl acetate elution through silica was found to display a broad-spectrum antimicrobial activity against opportunistic pathogens of the genus *Staphylococcus*, *Streptococcus*, *Enterococcus*, *Pseudomonas*, *Proteus*, *Stenotrophomonas*, and *Chromobacterium* with MIC and MBC values ranging from 0.0391 to 5 mg/mL. Biofilms of *S. aureus* MRSA ATCC 33593 and *S. aureus* MRSA NCTC 10442 were found to be susceptible to extract E3 with MBEC values of 1.25 mg/mL and 5 mg/mL respectively. Extract E3 failed to display toxicity against *G. mellonella* larvae up to and including the highest concentration of extract tested (20 μL of 16 mg/mL working solution) over a period of six days with 100% survival. Although the antimicrobial efficacy remains to be tested *in vivo*, the results suggest the presence of compounds that could be used against the emerging cystic fibrosis pathogen *Stenotrophomonas maltophilia* [85,86] or in a treatment strategy for *Staphylococcal* biofilm-related infections. 
